# Integrated analysis of mRNA and viral miRNAs in the kidney of *Carassius auratus gibelio* response to cyprinid herpesvirus 2

**DOI:** 10.1038/s41598-017-14217-y

**Published:** 2017-10-23

**Authors:** Jianfei Lu, Dan Xu, Yousheng Jiang, Shanyun Kong, Zhaoyuan Shen, Siyao Xia, Liqun Lu

**Affiliations:** 1 National Pathogen Collection Center for Aquatic Animals, Shanghai Ocean University, Shanghai, P. R. China; 2 Key Laboratory of Agriculture Ministry for Freshwater Aquatic Genetic Resources, Shanghai Ocean University, Shanghai, P. R. China; 3 National Experimental Teaching Demonstration Center for Fishery Sciences, Shanghai Ocean University, Shanghai, P. R. China

## Abstract

MicroRNAs (miRNAs) are small, non-coding single stranded RNAs that play crucial roles in numerous biological processes. Vertebrate herpesviruses encode multiple viral miRNAs that modulate host and viral genes. However, the roles of viral miRNAs in lower vertebrates have not been fully determined. Here, we used high-throughput sequencing to analyse the miRNA and mRNA expression profiles of *Carassius auratus gibelio* in response to infection by cyprinid herpesvirus 2 (CyHV-2). RNA sequencing obtained 26,664 assembled transcripts, including 2,912 differentially expressed genes. Based on small RNA sequencing and secondary structure predictions, we identified 17 CyHV-2 encoded miRNAs, among which 14 were validated by stem-loop quantitative real-time reverse transcription polymerase chain reaction (qRT-PCR) and eight were validated by northern blotting. Furthermore, Gene Ontology (GO) and Kyoto Encyclopedia of Genes and Genomes (KEGG) pathway analysis of miRNAs-mRNA pairs revealed diverse affected immune signalling pathways, including the RIG-I-like receptor and JAK-STAT pathways. Finally, we presented four genes involved in RIG-I-like pathways, including host gene IRF3, RBMX, PIN1, viral gene ORF4, which are negatively regulated by CyHV-2 encoded miRNA miR-C4. The present study is the first to provide a comprehensive overview of viral miRNA-mRNA co-regulation, which might have a key role in controlling post-transcriptomic regulation during CyHV-2 infection.

## Introduction

The alloherpesviridae family contains viruses that infect lower vertebrates, such as amphibians and fish, and many members infect important economically fish species^1^. Alloherpesviridae are very distantly related to the herpesviridae, with few genes being conserved between them^2–4^. Despite their evolutionary divergence, the families have many similar biological characteristics^1,2^. Cyprinid Herpesvirus-2 (CyHV-2) is a highly pathogenic member of the alloherpesviridae that causes acute mass mortality in populations of silver crucian carp (*Carassius auratus gibelio*) and goldfish (*Carassius auratus auratus*), via a condition referred to as herpesviral hematopoietic necrosis (HVHN)^1^. CyHV-2 has been reported as the pathogen responsible for mass mortality of silver crucian carp in China^5^. Additionally, epizootics caused by CyHV-2 infection have been reported in juvenile goldfish in many countries, including Asia^6^, Europe^7–9^, North America^10^, and Oceania^11^. Therefore, strategies to control CyHV-2 infections are required urgently to reduce the serious losses to aquaculture caused by this viral disease.

MicroRNAs (miRNAs) are a class of small non-coding RNAs involved in post-transcriptional regulation of target genes^12–14^. MiRNAs are transcribed initially in the nucleus as long primary miRNA precursors (pri-miRNA), which include one or several miRNAs embedded in the stems of ~80 nt long stem-loop structures^15^. The nuclear RNase III enzyme Drosha combines with these stem-loops and cleaves the stem at ~22 nt from the terminal loop to release a ~60-nt hairpin RNA, termed the pre-miRNA intermediate^16^. After export to the cytoplasm^14^, the pre-miRNA hairpin is further processed by a second RNase III enzyme, Dicer, to release the ~22-bp mature double-stranded miRNA duplex^17^. A single strand of the mature miRNA is incorporated subsequently into a cellular Argonaute (Ago) protein to form the RNA-induced silencing complex, RISC^18–20^; the other strand is degraded. Once incorporated into the RISC, the miRNA acts as a guide RNA to pair with specific target messenger RNAs bearing partially or fully complementary target sites, resulting in gene specific downregulation, either through enhanced translational inhibition or transcript degradation^21^. The miRNA target sites are usually located in the mRNA’s 3′ untranslated region (UTR), and while an miRNA’s association with the target mRNAs 3′ UTR does not need to be extensive, full complementarity to 2–7 or 8 bp of the miRNA, called the miRNA seed sequence, is generally required for effective downregulation^22^.

A number of herpesviruses, including the pathogen of common carp Cyprinid Herpesvirus 3 (CyHV-3), express virus encoded miRNAs in infected cells or i*n vivo* ^23,24^. Viral miRNAs play a key role in suppressing the expression of host cellular mRNAs, which often encode antiviral factors, as well as regulating the expression of viral genes, including crucial factors involved in the latent-to-lytic transition in viral infection^25,26^. In addition, viral miRNAs can target viral mRNAs to trigger their downregulation. For example, MR5057-miR-3p, encoded by CyHV-3, targets the 3′ UTR of ORF123, resulting in a reduced level of CyHV-3 dUTPase^23^. Additionally, miR-S1–5p and miR-S1-3p are encoded by the same miRNA precursor of polyomavirus SV40, and direct the cleavage of early transcripts during infection and regulate viral T-antigen transcripts negatively^27^. The reduction in the T antigen effected by miR-S1-5p and miR-S1-3p is crucial for the replication of the virus, causing a decrease in the numbers of SV40-specific cytotoxic T lymphocytes in T cells^27^. Human cytomegalovirus encoded miRNA miR-UL112-1 downregulates the expression of the cytomegalovirus gene IE1, which plays a crucial role in establishing latent infection^28^. In summary, the literature indicates that viruses have evolved to make the use of virus-encoded miRNAs to regulate the expression level of their own genes for successful infection.

In addition to “autoregulation” of viral target genes, several virus encoded miRNAs target host cellular mRNA^29^. However, their functions are poorly understood. Host cellular gene thrombospondin 1 (*THBS1*) is targeted by Kaposi’s sarcoma associated herpesvirus (KSHV) miRNAs. THBS1 has functions in downregulating angiogenesis and the growth of cells by promoting transforming growth factor beta (TGF)^30^. The downregulation of *THBS1* expression by KSHV miRNAs activates the survival and proliferation of KSHV-infected cells^30^. MiR-UL112-1, encoded by human cytomegalovirus, targets viral^31,32^ and host cellular genes^33^. Through binding with the 3′ UTR of the major histocompatibility complex class 1-related chain B gene (*MICB*), miR-UL112-1 inhibits the expression of *MICB* and further decreases the susceptibility of virus-infected cells to killing by natural killer cells^33^. Epstein barr virus (EBV) encodes miR-BHRF1–3, which downregulates the expression of CXC-chemokine ligand 11 (*CXCL11*), an interferon-inducible T-cell chemoattractant that plays a crucial role in host defences against EBV. Cells take advantage of the suppression of *CXCL11* to avoid T-cell recognition^34^. In recent years, virus-encoded miRNAs have attracted much research attention. However, many viral miRNAs have only been characterized in cell lines, and the roles of viral miRNAs in hosts *in vivo* may be very different to those in cell lines.

In lower vertebrates, the kidney, with the highest concentration of developing B lymphoid cells, is an important organ involved in adaptive immunity^35^. Additionally, CyHV-2 propagates most efficiently in the kidney, with the highest degree of tissue damage^36,37^. In this study, the viral miRNAs encoded by CyHV-2 were characterized in the kidney of *Carassius auratus gibelio*. We used high-throughput sequencing technology to analyse viral miRNA and mRNA expression profiles of *Carassius auratus gibelio* in response to CyHV-2. Based on small RNA (sRNA) sequencing and secondary structure predictions, we identified 17 CyHV-2 encoded miRNAs. Gene ontology (GO) and Kyoto Encyclopedia of Genes and Genomes (KEGG) pathway analysis of the miRNAs-mRNA pairs revealed the affected in immune signalling pathways. Finally, we presented a functional analysis of ORF4, ORF6, and *IRF3*, *RBMX*, and *PIN1*, which are targeted by miR-C4. Collectively, the present study is the first to provide a comprehensive overview of the viral miRNA-mRNA co-regulation, which might have a key role in controlling post-transcriptomic regulation during CyHV-2 infection.

## Results

### Overview of the deep sequencing of mRNA libraries

Expression profiling of the kidney of the control (T1K, T2K, and T3K) and moribund fish (T3K, T4K, and T5K) was carried using digital gene expression tag profiling (DGE). The major characteristics of these libraries are summarized in Table S1. A total of 573,340,100 raw reads were generated in the uninfected and infected groups. After filtering out the low quality reads, 564,633,312 clean reads remained. All clean reads were then assembled using the de novo assembly program Trinity^38^. The clean reads were assembled into 26,664 transcripts with an average length of 744 bp. The length distribution of these transcripts ranged from 201 to 14,287 bp. All the raw RNA-Seq data were submitted to the NCBI database (http://www.ncbi.nlm.nih.gov/geo/info/linking.html.) under accession number GSE90626.

To compare the mRNA expression profile between the CyHV-2 pre- and post-infected in silver crucian carp kidney, the sequence data were analysed using the DEGseq software, with the criteria of fold changes >2 and false discovery rate (FDR) <0.001, to identify significantly differentially expressed genes. The expressions of 2912 genes changed significantly, including 1422 upregulated and 1490 downregulated genes (Table S4). Subsequently, GO and KEGG enrichment analysis were performed to analyse the functions of the genes that responded to CyHV-2 infection. The significantly enriched GO terms included extracellular region, plasma membrane, integral to membrane, heme binding, electron carrier activity, and proteasome complex (Fig. S1). Pathway enrichment analysis for the differentially expressed genes showed that the proteasome, neuroactive ligand-receptor interaction, calcium signalling pathway, and PPAR signalling pathways were enriched (Fig. S2).

### CyHV-2 encodes multiple miRNAs that are clustered in distinct regions of the viral genome

We also identified CyHV-2-encoded miRNAs. RNAs isolated from the kidney of moribund and healthy fish were analysed using next generation sequencing (NGS). A total of 10,714,657 reads were generated in the uninfected and infected groups, with over 90% of the sequences being valid reads (Table S2). Among them, about 4.02% of the sequences mapping to Rfam, and most of the sequences were within 20 to 24 nucleotides in length. The remainder of the unmapped sequences likely represented non-coding RNAs, unrecognized miRNAs, or RNA degradation products.

**Figure 1:**
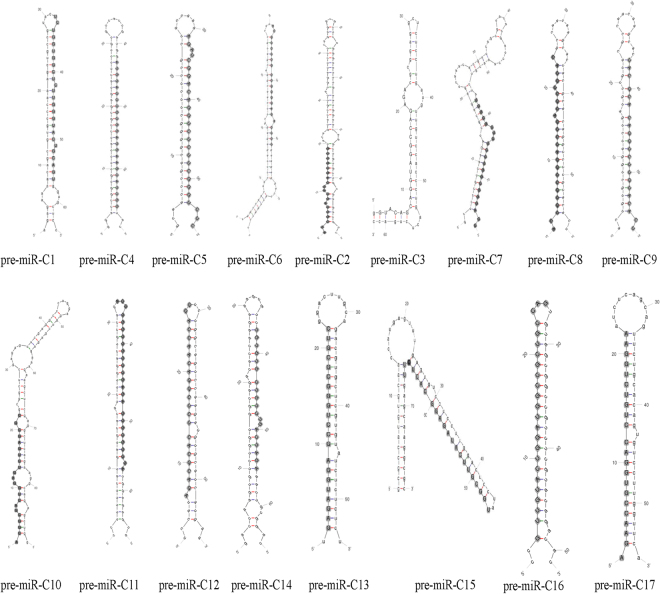
CyHV-2 pre-miRNA stem-loop structures. The stem-loop structures were predicted using the mfold program. Mature miRNA sequences are highlighted in grey and depicted in capital letters.

Seventeen potential CyHV-2 miRNAs were identified by NGS, based on sequence length, copy number, mapping to the CyHV-2 genome, and formation of stable hairpins. Secondary structure predictions^39^ demonstrated that the potential CyHV-2 miRNAs could fold into the hairpin structures typical of pre-miRNAs (Fig. 1). Table 1 shows the viral miRNAs, which ranged in size from 19 to 25 nucleotides and were detected at copy numbers from 3 to 417576. However, within the predominant consensus sequence for each miRNA, variability in the 3′ end of the miRNAs was common, as shown in Table S5, which lists all potential CyHV-2-derived miRNA and miRNA passenger strand reads. These miRNAs exhibited fewer sequence variations at the 5′ ends, while variations at the 3′ ends are fairly common. Similar 3′ and 5′ variability has been noted for many other herpesviruses^40–43^. Thus, in subsequent discussion, we considered the most abundant of all the isomiRs as the reference mature miRNA. Furthermore, miRNAs are generally not conserved between different viral species. Instances of conservation or high sequence similarity have only been observed between closely related viruses. We used BLASTN to align the putative CyHV-3 miRNA sequences to 17 CyHV-2 encoded miRNA; however, no homologous miRNAs were identified.

**Table 1:** Sequences and genome locations of CyHV-2 miRNAs.

miRNA name	miRNA_seq	miRNA length	strand	start	end	T1K read count	T2K read count	T3K read count	T4K read count	T5K read count	T6K read count
miR-C1	TCTGGTGGCTCTTGGGTACTGCAGT	25	−	5575	5596	0	0	0	0	3	2
miR-C2	ATCGTCATCATCAGCGTCG	19	+	15614	15633	0	0	0	5	5	6
miR-C3	GTACAGCAGGTAGGCCAGAGA	21	+	59934	59955	0	0	0	3	0	2
miR-C4	TGTTTTATCCGCGAGTACTTT	21	−	60757	60778	0	0	0	462	359	965
miR-C5	ATGTACCCGCGGATGAAGCATC	22	−	60825	60847	0	0	0	147148	277395	417576
miR-C6	ACGTCTCGCCGGGGAGACTCT	21	−	77087	77108	0	0	0	6	13	23
miR-C7	TTGCGCTCTCTTGGCGGGACC	21	−	81099	81120	0	0	0	1	6	6
miR-C8	ACTGTGCTGGATCTGATGCTGTAC	24	−	89662	89685	0	0	0	0	5	3
miR-C9	TACGCACAGGTCTTGTACACT	21	−	89698	179486	0	0	0	3	6	8
miR-C10	ACGGCGACTGGAGTCTGAGCGC	22	+	104014	104036	0	0	0	3	6	10
miR-C11	ACCTGGTTGTCCGAGAGTGCGTCTA	25	+	150379	150404	0	0	0	4	8	3
miR-C12	AGACGCACTCTCGGACAACCAGG	23	−	150391	150414	0	0	0	1	3	8
miR-C13	GAGATGGAGCCTGGGCGCGTC	21	+	180795	180816	0	0	0	1	4	6
miR-C14	TCTGTGCTGTCTGACTAGA	19	+	182236	182255	0	0	0	2	5	10
miR-C15	TGGCGTTCATAGAGGCACTCTT	22	+	194955	194977	0	0	0	8	3	11
miR-C16	GTTGTACTGGATGGCCGCTGCCAG	24	+	196075	196099	0	0	0	1	0	3
miR-C17	AGAACCGTGGACCTGTCTGGAA	22	+	208190	208212	0	0	0	4	2	7

**Figure 2:**
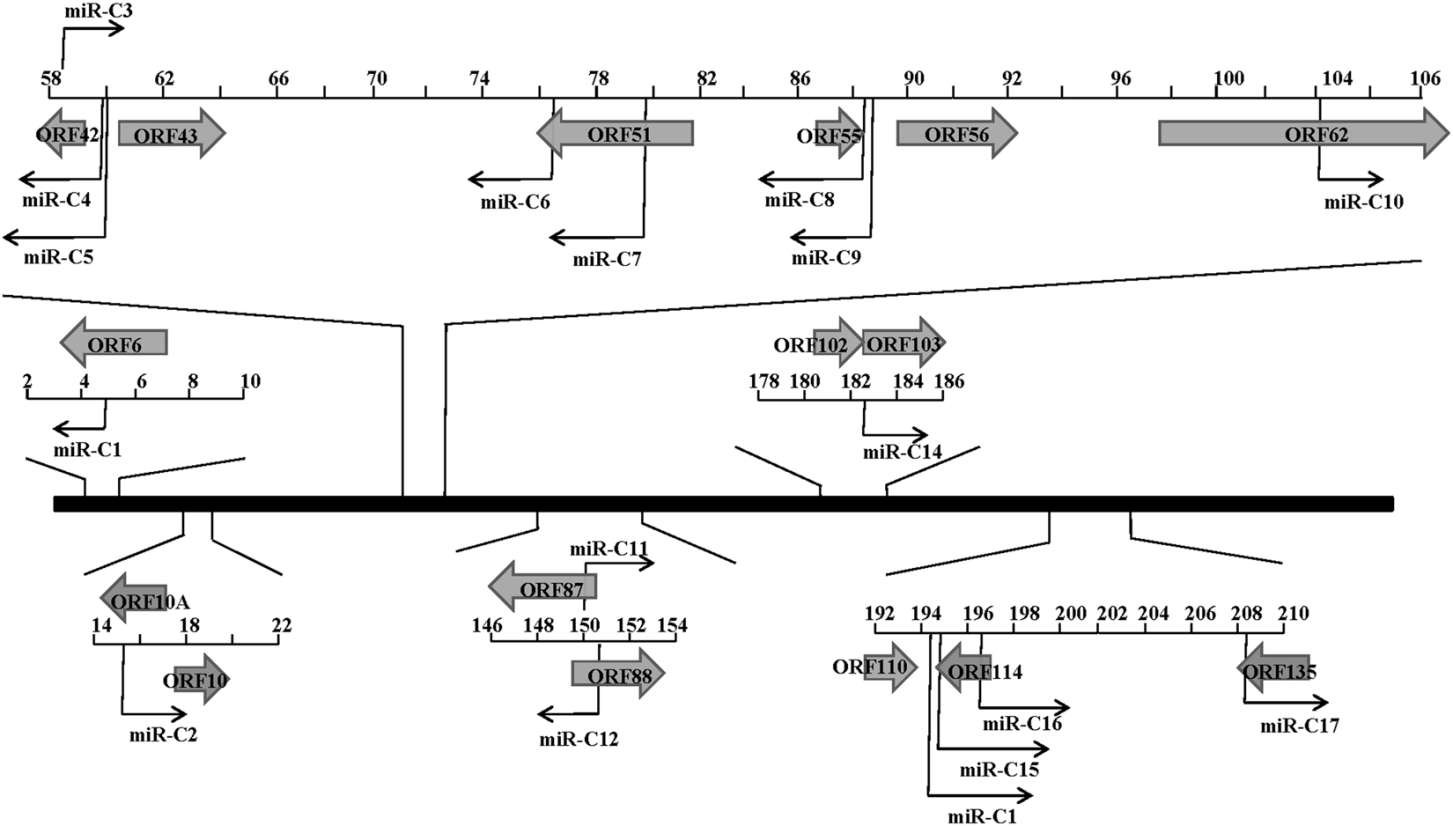
Genomic locations of CyHV-2 miRNAs. The CyHV-2 genome in the prototype orientation is shown in the middle as a thick black line. The CyHV-2 miRNAs are depicted as black arrows. Right-facing arrows indicate miRNAs encoded on the sense strand, while the left-pointing arrows indicate miRNAs encoded on the antisense strand. Large grey arrows represent annotated open reading frames.

The genomic locations of the 17 potential CyHV-2 miRNAs are depicted in Fig. 2. Similar to many other herpesviruses, the CyHV-2 miRNAs are generally distributed across the viral genome, with major clusters in two different regions. One cluster of seven miRNAs is located near ORF42, including miR-C3, miR-C4, and miR-C5. A second cluster exists near ORF114, including miR-C1, miR-C15, and miR-C16. Noticeably, two pre-miRNAs encode two different mature miRNAs, one of which was on the 5′ end and the other was on the 3′ end, including miR-C8 with miR-C9, and miR-C11 with miR-C12. Detailed sequences, loci, read numbers, and orientations of these miRNAs are shown in Fig. 2. The most abundant miRNA was miR-C5, and the second most abundant miRNA was miR-C4.

### Detection of CyHV-2 miRNAs by northern blotting and stem-Loop quantitative real-time reverse transcription polymerase chain reaction (qRT-PCR) following CyHV-2 infection

**Figure 3:**
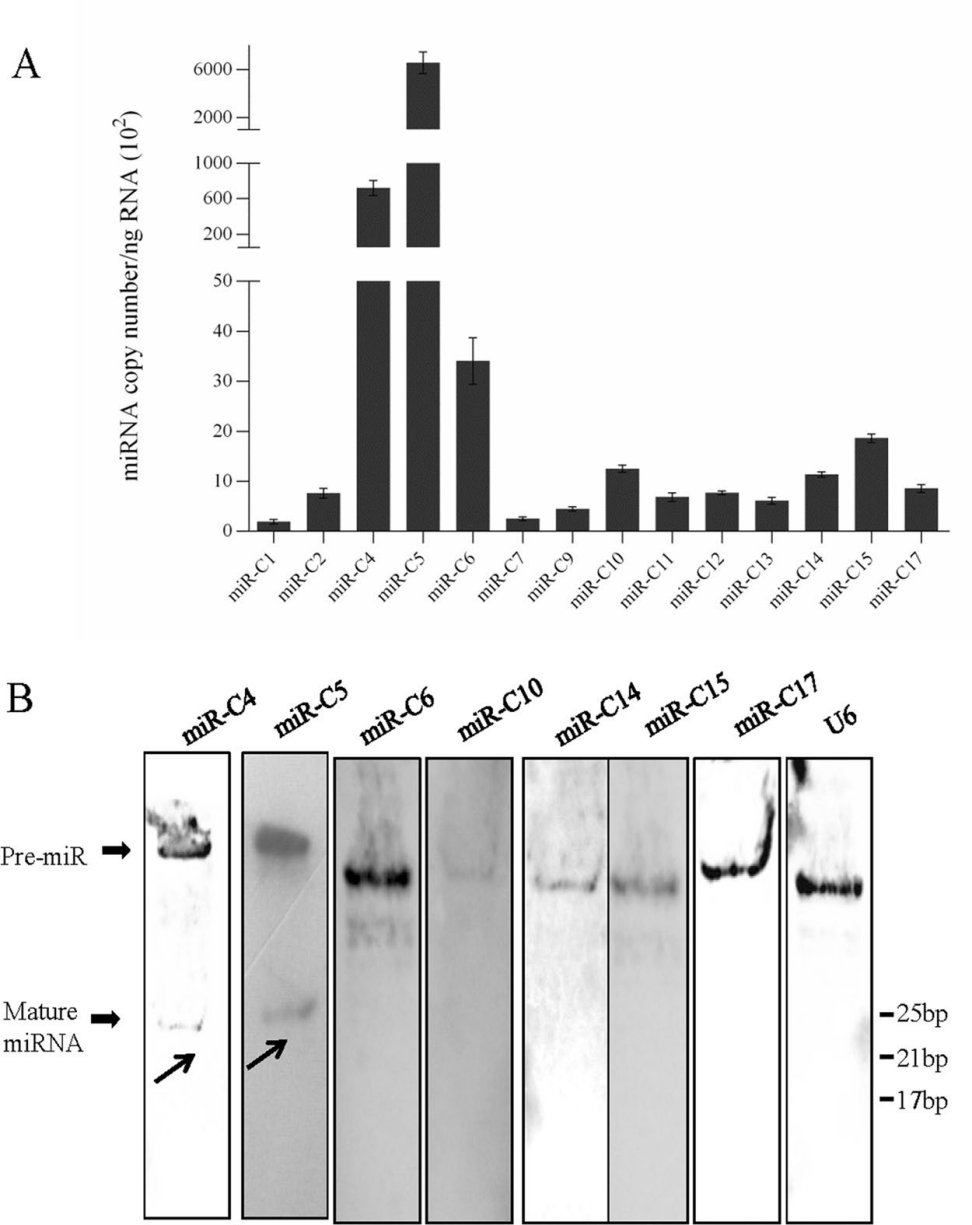
Verification of CyHV-2 encoded miRNAs. (**A**) Stem-loop qRT-PCR of the seventeen CyHV-2 miRNAs among total RNAs isolated from kidney organs of CyHV-2 moribund fish. The data were obtained from three independent experiments (mean ± SD). (**B**) Verification of CyHV-2 encoded miRNAs. Total RNAs prepared from kidneys of moribund fish were blotted with DIG-labelled oligodeoxynucleotide probes. U6 was used as a loading control, and the mature miRNA are indicated as black arrows.

Stem-loop qRT-PCR is a sensitive technique to detect mature miRNAs specifically^44^. Therefore, it was used to test the CyHV-2 miRNA from the infected *Carassius auratus gibelio* kidney. Custom Hairpin-it™ MicroRNAs Quantitation PCR Kits were designed to detect the mature miRNAs. Using these assays, the target miRNAs were detected in the CyHV-2 infected sample. We were able to detect 14 mature miRNAs reliably, but not miR-C3, miR-C8 and miR-C16. The expression of CyHV-2 miRNAs showed two distinct expression patterns. The first pattern was demonstrated by miR-C4 and miR-C5, which were the most abundant. Similarly, miR-C1, miR-C2, miR-C6, miR-C7, miR-C9, miR-C10, miR-C11, miR-C12, miR-C13, miR-C14, miR-C15 and miR-C17 also accumulated, but were much less abundant (Fig. 3A).

Viral miRNAs displaying higher expression levels were selected for northern blotting analysis. The pre-miRNAs of miR-C4, miR-C5, miR-C6, miR-C10, miR-C14, miR-C15, and miR-C17 were detected, and mature miRNA of miR-C4 and miR-C5 were detected by northern blotting. The blot pattern showed two bands for pre-miR-C6, indicating that pre-miR-C6 might be generated by different mechanisms (Fig. 3B).

### Identification of miRNA-mRNA regulatory interactions associated with CyHV-2 infection

Increasing evidence shows that viral miRNAs play crucial roles in host-virus interactions, and many studies have demonstrated that viral miRNAs can target viral or host genes^45^. Systematic analyses of the interactions between mRNA and miRNA could reveal information concerning the roles of miRNAs during virus infection^46^. In the present study, targets of miRNAs were identified based on sequence complementarities and the free energy of the predicted RNA duplex using miRanda and TargetScan. A total of 1108 miRNA-mRNA interactions were identified. The predictions showed that most miRNAs could regulate several target genes. For example, miR-C4 correlated with two viral genes and 70 host genes, and miR-C3 correlated with 174 host genes and five viral genes. Moreover, most mRNAs are associated with more than one miRNA, such as host genes caspase 8, which was targeted by miR-C8, miR-C12, and miR-C14; and carboxypeptidase D, which was targeted by miR-C8, miR-C13, and miR-C14 (Table S6).

To detect the functional characteristics of the miRNA-mRNA interaction pairs, the mRNAs involved in interaction pairs were subjected to a GO and KEGG pathway analysis. GO analysis provided insight into the functions of genes in various biological processes^47^. Based on the GO functional analysis, the targets of the miRNAs were enriched in cell redox homeostasis, Rho GDP-dissociation inhibitor activity, nucleoside phosphate kinase activity, and thyroid gland development (Table S7, Fig. S3). In organisms, genes often interact with each other to exert their different roles in certain biological functions^48^. KEGG pathway analysis could aid our understanding of the biological functions of genes^49,50^. In the KEGG enrichment classification, Type II diabetes mellitus, the JAK-STAT signalling pathway, bacterial invasion of epithelial cells, and the RIG-I-like receptor signalling pathway were involved in the significantly enriched miRNA-associated pathways (Table S8, Fig. S4). We also noted several immune-related pathways that were significantly enriched, including the regulation of the RIG-I-like receptor signalling pathway (Table S8), which suggested the important role of CyHV-2 miRNAs in restricting innate antiviral immunity. Thus, GO and KEGG analyses provide a better understanding of the cellular components, molecular functions, and biological processes of target genes, and provided a reference for future research.

To narrow the focus of our study to viral miRNA target genes that are relevant to anti-virus immunity, we analysed the RIG-I-like pathway, which plays a crucial role in the host innate immune responses against viral pathogen infections. In our study, 15 RIG-I-like receptor pathway-related genes were found to be differentially expressed, and three of these genes were regulated by miR-C4 (Table S9).

### Detection of the level of CyHV-2 and the DEGs in the kidney of silver crucian carp

**Figure 4:**
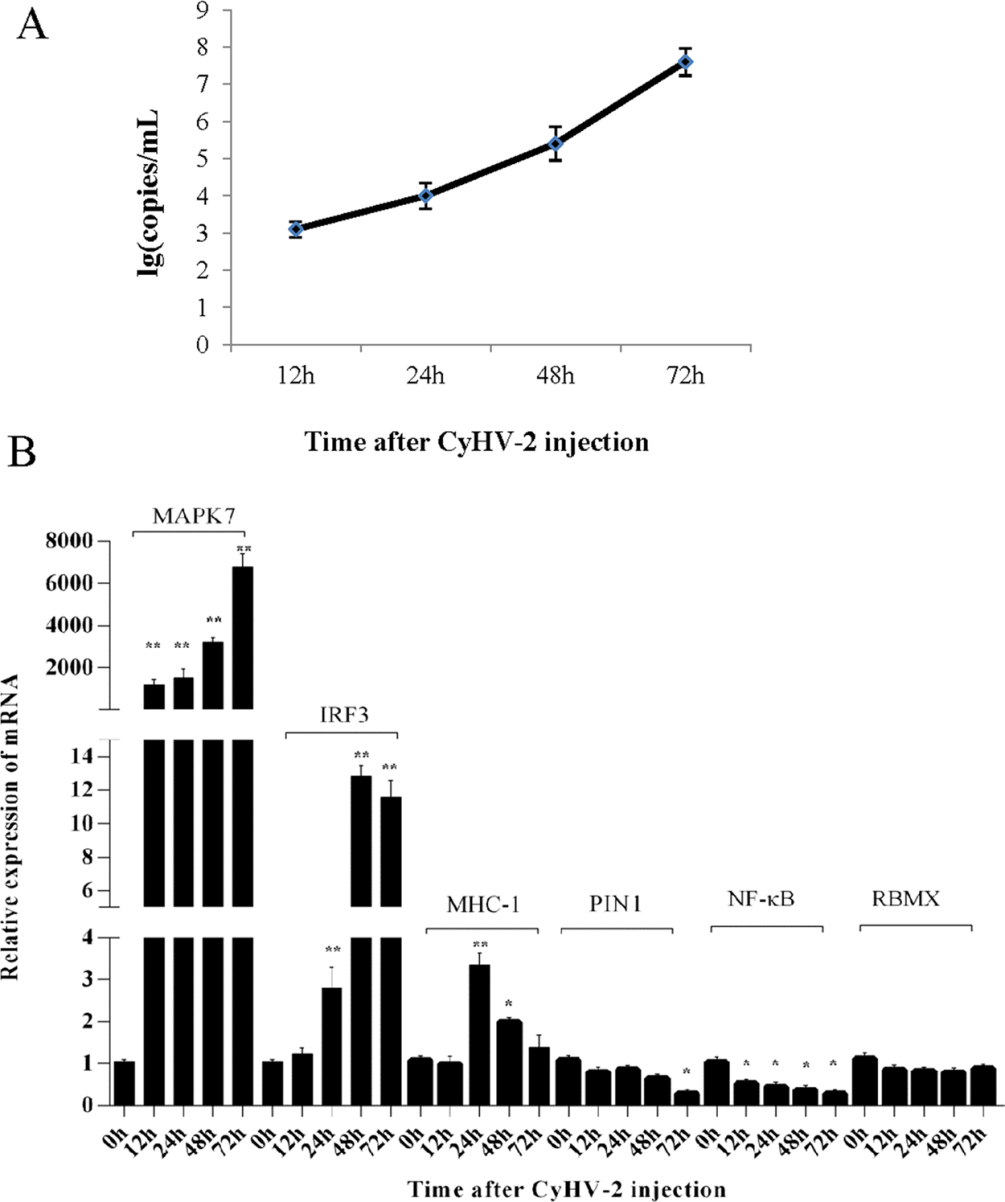
The level of CyHV-2 and the DEGs in the kidney of silver crucian carp. Samples were collected at 0, 12, 24, 48, and 72 h after challenged with CyHV-2. (**A**) The accumulation curve of CyHV-2 in the kidney of silver crucian carp. Viruses were titrated using a real-time PCR approach, as described previously^5^. (**B**) The relative expression levels of *MAPK7*, *MHC-I*, and *NF- κB*; (**C**) *IRF3*, RBMX, and PIN1 in the kidney, β-actin served as the reference gene. Results are presented as mean ± SD of 3 independent experiments, *P < 0.05, **P < 0.01.

The quantitative RT-PCR for CyHV-2 titration was performed as described previously^5^. qRT-PCR assays showed that the virus titres increased over time, and reached 10^7.6^ at 72 h post infection (Fig. 4A). In addition, to validate the NGS data, certain differentially expressed genes were confirmed by qRT-PCR, including *IRF3*, *RBMX*, *PIN1*, *MAPK7*, *MHC-I*, and *NF- κB*. As shown in Fig. 4B, *PIN1* and *NF- κB* mRNA levels were significantly downregulated after the infection, *MHC-I*, *IRF3*, and *MAPK7* expressions were significantly upregulated following infection, whereas *RMBX* expression showed almost no variation.

### Validation of miRNA targets by luciferase activity

**Figure 5:**
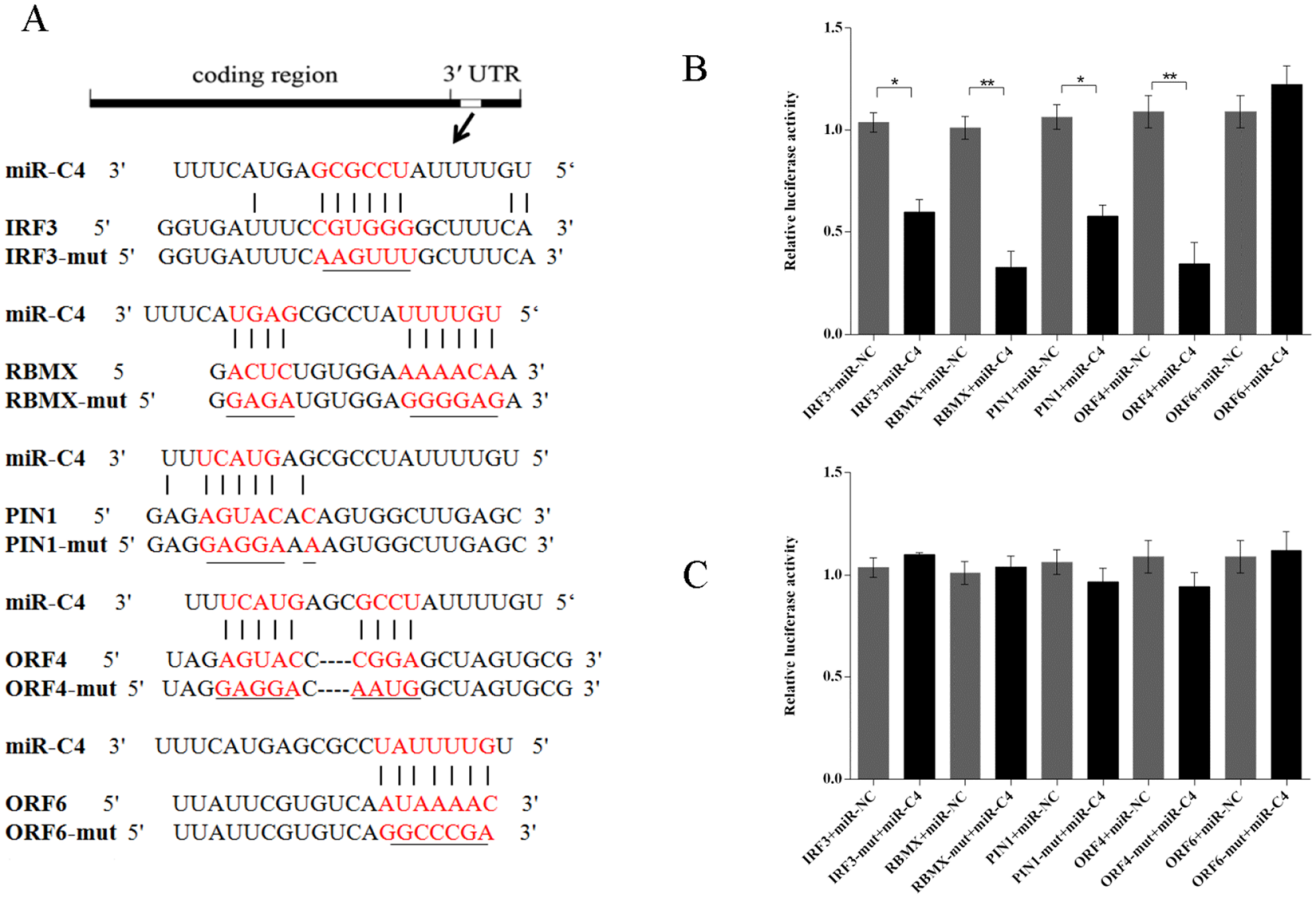
Regulation of *IRF3*, *RBMX*, *PIN1*, ORF4, and ORF6 by miR-C4. (**A**) The alignment between miR-C4 and the 3′ UTR segment of *IRF3*, *RBMX*, *PIN1*, ORF4, and ORF6. The sequences of the mutated target sites are shown below the alignments in each panel. Mutated nucleotides are underlined. (**B**) Luciferase activity in Hela cells transfected with miRNA mimics and plasmids carrying the 3′ UTR of I *IRF3*, *RBMX*, *PIN1*, ORF4, and ORF6. NC miRNA: negative control miRNA. (**C**) Luciferase activity in Hela cells transfected with miRNA mimics and plasmids carrying the mutant 3′ UTR of *IRF3*, *RBMX*, *PIN1*, ORF4, and ORF6. The data were obtained from three independent experiments (mean ± SD). *P < 0.05, **P < 0.05.

To reveal the pathways mediated by viral miRNAs, the target genes of viral miRNAs that are involved in the RIG-I-like pathway were analysed. Previous research revealed that viral miRNAs are regulators of the networks involved in regulating the RIG-I-like pathway^51^. Based on target prediction using TargetScan and miRanda, CyHV-2-encoded ORF4 and ORF6, and host gene *IRF3*, *RBMX*, and *PIN1* genes were identified as the targets of miR-C4 (Table S9), and *IRF3*, *RBMX*, and *PIN1* are involved in the RIG-I-like pathway. The binding sites of miR-C4 to ORF4, ORF6, *IRF3*, *RBMX*, and *PIN1* in *Carassius auratus gibelio* are shown in Fig. 5A.

To evaluate the functions of miR-C4 on target 3′ UTRs, dual-luciferase reporter constructs carrying wild-type or mutant ORF4, ORF6, *IRF3*, *RBMX*, and *PIN1* 3′ UTRs were cotransfected with miR-C4 mimics or negative control miRNA (a random miRNA sequence). The results showed that the luciferase activities of ORF4, *IRF3*, *RBMX*, and *PIN1*were significantly reduced by the miR-C4 mimics, but did not affect the mutant reporter (Fig. 5B,C). This result indicated that miR-C4 targets a sequence in the 3′ UTR of ORF4, *IRF3*, *RBMX*, and *PIN1*.

## Discussion

In recent years, many herpesviruses have been found to encode miRNAs, including the pathogenic virus of common carp, CyHV-3. The aim of this study was to analyse the mRNA targetomes regulated by CyHV-2 encoded miRNAs, a closely related virus to CyHV-3. In the present study, we used NGS to characterize mRNAs and miRNAs in CyHV-2 infected *Carassius auratus gibelio* kidneys. Seventeen CyHV-2 miRNAs were identified, and fourteen were confirmed by stem-loop qRT-PCR. We performed an integrative analysis of these data and obtained the complete set of CyHV-2 encoded miRNAs and host genes.

The copy number of viral miRNA reads were quite low in many viruses^23,52,53^. The factors that contribute to miRNA abundance include the amount of transcript accumulation, the efficiency of miRNA processing, and the contribution of miRNA decay. Considering that almost all the 156 CyHV-2 ORFs are transcribed during infection, it is unsurprising that the majority of CyHV-2 transcripts sequenced represented mRNA degradation products. By comparison, fewer CyHV-2 miRNA reads mapped to non-coding regions; however, these were much more abundant in terms of read count. Additionally, miRNA stability can be influenced by cellular modifications, argonaute protein levels, exposure of the miRNAs to nucleases, and target abundance^54^. EBV miRNAs were documented to be differentially expressed in distinct cultured cell types, similar to observations in many studies of cellular miRNAs^55^. Some investigations revealed that four of the 24 miRNAs encoded by the rhesus cytomegalovirus (RhCMV) were detected exclusively in infected fibroblasts, while two were specific for infected salivary glands^42^. Thus, the distribution of CyHV-2 miRNAs may be regulated in a tissue-specific manner *in vivo*. In our study, the CyHV-2 miRNAs identified were detected by both NGS and qRT-PCR. In the NGS and qRT-PCR assays, miR-C4 and miR-C5 were the most abundant microRNAs during infection, and the remaining viral miRNAs were much less abundant (Fig. 3A, Table 1). The different abundance of CyHV-2 miRNAs enriched in the kidney of infected silver crucian carp suggested that these miRNAs might be regulated in a tissue-specific manner *in vivo*. Additionally, the high abundance of miR-C4 and miR-C5 suggested significant roles for these miRNAs during infection.

It has been reported that viral miRNAs can target and downregulate host cellular mRNAs and/or viral mRNAs during virus infection^56–59^. Exploiting viral miRNA targets from the host or viral mRNA enabled us to screen for important candidate targets of CyHV-2 miRNAs, which indicated their roles in evading the host innate immune responses, such as antiviral signalling, inflammation, and apoptosis. The innate immune system in fish is regarded as the first line of defence against pathogens and is much more important in fish than in mammals^60^. The JAK/STAT signalling pathway has been demonstrated to play an important role in the antiviral response of vertebrate^61^. However, the regulation of JAK/STAT transcription factor expression mediated by viral miRNAs has not been investigated^62^. The measles virus (MV) phosphoprotein can combine with the linker domain of signal transducer and activator of transcription 1 (STAT1), resulting in inhibit the JAK/STAT activation^63^. The Hepatitis C virus (HCV) core protein is required for the infectious viruses production through interaction with the JAK protein^64^. In this study, target prediction indicated that the viral miRNAs could target genes involved in the JAK-STAT pathway, including *JAK1*, *STAT1*, *STAT6*, and *MYC1* (Table S8). The findings indicated a novel aspect of viral miRNA-mediated JAK/STAT signalling pathway regulation during virus infection.

During infection, virus encoded miRNAs regulate the RIG-I antiviral pathway and host immune response. The results presented by Silva and Jones^65^ suggested that the expression and production of the BHV-1-infected cell protein 0 (bICP0) is interfered with by Bovine Herpes Virus1 (BHV-1) miRNAs, which are expressed during latent infection and stimulated the RIG-I signalling pathway, which correlated with activated type I interferon signalling. However, they presented no valid evidence of the mechanism of how the latency-related gene-encoded miRNAs were recognized by RIG-I. In this study, we identified certain genes involved in the RIG-I-like pathway, including *IRF3*, *RBMX*, and *PIN1*, which are negative regulated by CyHV-2 miRNA miR-C4 (Table S6). In addition, validation of the miRNA-mRNA interaction pairs showed that *PIN1* had a down-down regulatory pattern and *IFR3* presented a down-up regulating pattern; *RMBX* expression hardly changed (Fig. 5B). This result mostly corresponded with the sequencing data. Extensive research has shown that hundreds of miRNAs interact with thousands of target mRNAs to maintain proper gene expression patterns under viral infection, and our results support this notion. The complexity of the miRNA-mRNA interaction network presents a great challenge for researchers to reveal the roles for specific miRNAs or miRNA-mRNA interactions in biological processes. For instance, *PIN1*, *IFR3*, and *RMBX* constituted a complex interaction network with miR-C4, miR-C12, and many other miRNAs (Table S6). Furthermore, miRNA-mRNA interaction is only one of the multiple mechanisms influencing the regulation of gene expression, and our results would not be sensitive in circumstances where multiple factors, in addition to miRNA-mRNA interactions, are involved. Although increasing evidence shows that viral miRNAs affect host innate immune responses to regulate virus infection^66^, the exact mechanisms of the roles of miRNAs in the host immune response to viral infection remain to be determined.

Overall, our results demonstrated a series of complex sequential viral miRNA molecular signatures associated with CyHV-2 infection and provided a basis for future investigations. We identified 17 CyHV-2 encoded miRNAs. GO and KEGG pathway analysis of the reported viral miRNAs revealed the diversity of the affected immune signalling pathways, including the RIG-I-like receptor pathway and the JAK-STAT pathway. The post-transcriptional regulation of IRF3, RBMX, PIN1, and ORF4 by miR-C4 could affect the expression of those genes.

## Materials and Methods

### Fish and CyHV-2 challenge

Healthy silver crucian carp (approximately 10 cm in body length) were obtained from the Wujiang National Farm of Chinese Four Family Carps, Jiangsu Province, China.

Initially, fish were temporarily reared at 23 °C for adaptation. After seven days of acclimation, fish were divided into two groups (30 fish per group) for intraperitoneal injection. The conditions were identical among the tanks and the fish were randomly distributed into the different tanks. Two groups were maintained in two aquariums and intraperitoneally injected with CyHV-2 suspended in PBS at a dose of 1 × 10^6^ TCID50/g, which was applied and verified by previous challenge experiments^5^. As controls, fish were injected with PBS at the same dosage. After injection, all the fish were reared under the same conditions, fed with a diet according to a standard feeding scheme, and observed continuously to identify and collect moribund animals. Moribund fish at 72 h post-challenge were collected, and kidneys of control fish (T1K, T2K, and T3K) and moribund fish (T4K, T5K, and T6K) were sampled, each comprising three biological replicates, and three different individual kidney tissues, and immediately frozen in liquid nitrogen. RNA samples were prepared for transcriptome and gene expression analyses. All experiments were performed according to the guidance of the Care and Use of Laboratory Animals in China. This study was approved by the Committee on the Ethics of Animal Experiments of Shanghai Ocean University, China.

**RNA isolation, library construction, and sequencing**. For the six transcriptome library constructions, the RNA preparation, library construction, and high-throughput sequencing were performed by LC-BIO (Hangzhou, China). The experimental procedure was as follows: total RNAs were extracted using the Trizol reagent (Invitrogen, CA, USA), following the manufacturer’s instructions. The quantity and purity of the total RNA were analysed using a Bioanalyzer 2100 and RNA 6000 Nano LabChip Kit (Agilent, CA, USA) and had an RNA integrity (RIN) number greater than 7.0. For the sRNA-seq experiment, approximately 1 μg of total RNA was used to construct an sRNA library, according to the protocol of the TruSeq™ Small RNA Sample Prep Kits (Illumina, San Diego, CA, USA). Single-end sequencing (50 bp) was performed on the Illumina Hiseq. 2500 platform following the vendor’s recommended protocol. For the RNA-seq experiment, approximately 10 μg of total RNA was subjected to enrichment for poly (A)-tailed mRNAs using poly-T oligo attached magnetic beads (Invitrogen, MA, USA). Following purification, the mRNAs were fragmented into small pieces using divalent cations at an elevated temperature. The cleaved RNA fragments were then reverse-transcribed to produce the final cDNA library, according to the protocol of the mRNA-seq sample preparation kit (Illumina, San Diego, CA, USA). The library was constructed by pooling nine homogenized total RNAs from the kidney samples. Then, paired-end sequencing of the libraries was performed on an Illumina Hiseq. 2500 (LC Sciences, USA), following the vendor’s recommended protocol. The length of the reads was 100 bp, and the average insert size for the paired-end libraries was 179 bp (the length of the adapter was 121 bp).

### Pre-treatment of the sRNA-seq data

Small RNA libraries were constructed and sequenced as previously described^67^. Total RNA used to make the small RNA library was prepare according to the manufacturer’s instructions of TruSeq Small RNA Sample Prep Kits (Illumina, San Diego, CA, USA). The sRNA libraries were then sequenced by Illumina Hiseq. 2500 50SE at the LC-BIO (Hangzhou, China). The raw reads were subjected to the Illumina pipeline filter (Solexa 0.3), and then the dataset was processed with ACGT101-miR (LC Sciences, Houston, TX, USA), to remove repeats, junk, adapter dimers, low complexity, and common RNA families (rRNA, tRNA, snRNA, and snoRNA). Subsequently, unique sequences of 19–25 nt were mapped to specific species miRNA precursors in miRBase 21.0 using Bowtie search to identify known and novel miRNAs. Subsequently, all the remaining sRNA sequences were searched against the CyHV-2 genome (GenBank accession no. AF332093.1). The small RNA sequences of putative CyHV-2 miRNAs were analyzed by a BLASTN search against the CyHV-2 genome, allowing one or two mismatches between each pair of sequences. To analyze the potential pre-cursor structures of 17 CyHV-2 miRNA candidates, each sequence, including a fragment of 60 to 70 bases flanking the sequence, was subjected to miRNA secondary structure prediction using the mFold online software (http://frontend.bioinfo.rpi.edu/applications/mfold/) with default parameters.

### De novo assembly and expression level calculation of the transcripts

The raw reads were cleaned by removing adapter sequences, empty reads, and low quality sequences (reads with over 10% unknown base pairs ‘N’). The reads obtained were randomly decomposed into overlapping k-mers (default k = 25) for assembly using the Trinity software^38^. After assembling the transcripts, a locally installed BLAST all program^68^ was used to search the assembled transcripts against the sequences in NCBI NR protein database (http://www.ncbi.nlm.nih.gov/protein/)^69^ and the Swissprot database (http://www.uniprot.org/)^70^ using an E-value cut-off of lower than 1e-10. Genes were tentatively annotated according to their best hits against known sequences. The clusters of orthologous groups (COG^71^) and KEGG^72^ annotation systems were used to analyse the biological pathways that the involved the assembled transcripts. Furthermore, Bowtie (version 0.12.7)^73–77^ was used to map RNA-seq reads to all the assembled transcripts using the “single-end” method and the parameter “-v 3 -a – phred 64-quals” (allowing one read to be mapped to multiple transcripts). The perfectly mapped read counts were retained for expression level calculation using the following formula: expression level of a transcript (RPKM: reads per kilobase of exon model per million mapped reads) = Number of the reads mapped to the transcript/[Total number of the reads mapped to all the transcripts (in million) × the length of the transcript (in kilobases)].

### The Prediction of miRNA Target Genes

miRNA target prediction algorithms TargetScan 50 (http://www.targetscan.org/) and miRanda3.3a (http://www.microrna.org) were used to identify miRNA binding sites. Finally, the data predicted by both algorithms were combined and the overlaps were calculated. GO terms and KEGG pathways of these miRNAs and miRNA targets were also annotated.

### Northern blotting

Total RNA was extracted from tissues using an miRNeasy Kit (Qiagen) according to the manufacturer’s instructions. Samples of 20 mg of total RNA were resolved using a 15% polyacrylamide gel containing 8 M urea and transferred to Hybond-N + nylon membrane (GE). After cross-linking with UV light, the membrane was pre-hybridized in DIG Easy Hyb granule buffer (Roche, Switzerland) for 30 min. Subsequently, the membrane was hybridized with a digoxigenin (DIG)-labelled DNA probe complementary to a specific miRNA sequence for 12 h at 40 °C. Signal detection was performed as described in the manual for a DIG High Prime DNA labelling and detection starter kit II (Roche, Switzerland).

### Validation of miRNA and mRNA expression by quantitative real-time reverse transcription polymerase chain reaction (qRT-PCR)

For mRNA quantification, PrimeScript™ RT Master Mix (Takara, Japan) was used to synthesize first-strand cDNA. qRT-PCR was performed with the SYBR Premix Ex Taq™ (Takara), using gene-specific primers for *IRF3*, *RBMX*, and *PIN1*, β-actin was used as an internal standard (Table S3), The 2^−ΔΔCT^ method was adopted to analyze the expression of the different genes. All the expression data were subjected to a one-way ANOVA, and statistical significance was assumed at P < 0.05.

For miRNA quantification, the Hairpin-it™ MicroRNAs Quantitation PCR Kit (GenePharma, China) was used to quantify mature miRNAs according to the manufacturer’s instructions. Total RNA was isolated from kidney organs of CyHV-2 infected fish, and 1 μg of total RNAs were used for cDNA synthesis. PCR amplification was performed in a 20 μL reaction containing 2 μL of cDNA, 10 μL of Real-time PCR Master Mix (FAM), 10 μM of miRNA specific Primer, and 10 μM of miRNA specific probe, 1U DNA polymerase. Synthetic miRNA (GenePharma, China) was used as the standard. Data was normalized to total RNA and used to determine the relative miRNA copy number per 1 μg of total RNA. Each reaction was performed in triplicate and the data were calculated as the mean ± SD as described above. All reactions were performed in triplicate on the CFX96 Real-time PCR Detection System (Bio-Rad, Hercules, CA, USA).

### Validation of miRNA targets by luciferase activity

The 3′ UTRs of ORF4, ORF6, *IRF3*, *RBMX*, and *PIN1*, containing miR-C4 binding sites, were amplified from silver crucian carp kidney cDNA, using the primers shown in Table S3. All the PCR products were cloned into the pGL3-Basic Dual-Luciferase miRNA Target Expression Vector (Promega, USA) via the Smal and Xhol restriction sites. The enzymes used for the cloning were purchased from Takara, China. Sangon (China) verified the DNA sequences of the constructs. Site-directed mutagenesis was performed on the 3′UTR reporter plasmid using the Fast Site-Directed Mutagenesis Kit (Tiangen, China), and the primers are shown in Table S3.

Hela cells was cultured in MEM Medium (Gibco, USA) supplemented with 10% foetal bovine serum (Gibco, USA), at 37 °C with 5% CO_2_. miRNA mimics (miR-C4/negative control, GenePharma, China) were transfected separately into the cell line with a luciferase reporter vector containing target genes using Lipofectamine 3000 reagent (Invitrogen, USA) in 96-well plates. Hela cells were incubated for 24 h after transfection, and then the Dual-Glo luciferase assay system (Promega, USA) and GloMax-Multi Detection System were used to detect the quantity of firefly and Renilla luciferase, respectively. The firefly luciferase activity was first normalized to the Renilla luciferase activity, and the ratios were then normalized to the levels in the empty vector controls.

## Electronic supplementary material

Supplementary Dataset
